# Effects of sweet almond (*Prunus amygdalus*) suspension on blood biochemical parameters in experimentally induced hyperlipidemic mice

**DOI:** 10.14202/vetworld.2019.1966-1969

**Published:** 2019-12-14

**Authors:** Afaf A. Tarmoos, Lubna A. Kafi

**Affiliations:** 1 Department of Physiology and Pharmacology, College of Veterinary Medicine, University of Baghdad, Baghdad, Iraq; 2 Department of Pharmacology, College of Medicine, Ibn Sina University of Medical and Pharmaceutical Sciences, Baghdad, Iraq

**Keywords:** coagulation factors, hyperlipidemia, lipid profile, mice, *Prunus amygdalus*, sweet almond

## Abstract

**Aim:**

The present study aimed to examine the effects of sweet almond (*Prunus amygdalus*) suspension (SAS) on the measurements of blood biochemical parameters in male albino mice, in which hyperlipidemia was induced experimentally.

**Materials and Methods:**

Seventy male albino mice were divided randomly into seven groups (10 mice/group). The first group was the untreated control group (negative control). The second group comprised hyperlipidemic mice that did not receive SAS treatment (positive control). The other five groups consisted of hyperlipidemic mice that were orally administered five different doses of SAS (285, 571, 857, 1128, and 1428 mg/kg body weight). Hyperlipidemia was induced in mice by adding 1% cholesterol to the diet along with 0.5% H_2_O_2_ to the drinking water, with *ad libitum* access to both food and water for 60 consecutive days. Prothrombin time, partial thromboplastin time, clotting time, and platelet count were measured. Serum lipid profile (total cholesterol [TC], triacylglycerol [TAG], low-density lipoprotein cholesterol [LDL-C], very LDL-C [VLDL-C], and high-density lipoprotein cholesterol [HDL-C]) was also determined.

**Results:**

Prothrombin time, partial thromboplastin time, and clotting time significantly increased only in groups treated with SAS, especially at the dosage of 1428 mg/kg compared with the positive control group. Blood platelet count significantly decreased in SAS-treated groups. The serum levels of TC, TAG, LDL-C, and VLDL-C in the SAS-treated groups (857, 1128, and 1428 mg/kg) significantly decreased, whereas the serum level of HDL-C significantly increased compared with that of the positive control group.

**Conclusion:**

SAS administered orally at 1428 mg/kg body weight was the dose that most significantly decreased platelet count and serum levels of TC, TAG, LDL-C, and VLDL-C and increased prothrombin time, partial thromboplastin time, and clotting time as well as serum level of HDL-C in experimentally induced hyperlipidemic mice.

## Introduction

Intake of excessive saturated fats and cholesterol leads to obesity, hyperlipidemia, and cardiovascular diseases such as myocardial infarction and arteriosclerosis, which causes cellular lipid peroxidation, enhances the occurrence of cancers and aging, and promotes biomembrane changes or destruction [1-3]. Herbal medicines or phytomedicines have been reported to modulate various disease conditions such as cancer [4,5], diabetes [6], and hyperlipidemia [7,8], affect the reproductive system in male rodents [9,10] and promote wound healing [11].

Aqueous sweet almond (*Prunus amygdalus*) suspension (SAS) has been found to be therapeutic in many cases of diabetes [6] and arthritis [12,13]. It was also reported to alter the coagulation process in mice [14]. In the previous studies conducted at our laboratory, we have reported the efficacy of aqueous and hexane extracts of sweet almond for treating hyperlipidemia in mice [7,8]. No prior data are available on the effects of SAS in hyperlipidemic mice. Hyperlipidemia is a metabolic disorder that can be induced in laboratory animal models such as rats and mice [3,7,8,15]. Dietary hyperlipidemia is usually accompanied by metabolic changes in the serum glucose levels [6] as well as changes in the blood picture [14]. The condition of hyperlipidemia can be induced in rodents through excessive dietary supply of cholesterol (1%) with concomitant administration of 0.5% H_2_O_2_ in the drinking water [7,8,15]. The potential therapeutic efficacy of sweet almond in hyperlipidemia needs extensive primary studies in experimental animals. Further, the dose-response effects of SAS in hyperlipidemia are still not clear, especially in laboratory mice.

The present study was undertaken to examine the effects of SAS on blood biochemical parameters in mice following the induction of hyperlipidemia by feeding a diet supplemented with excess cholesterol combined with H_2_O_2_ in the drinking water.

## Materials and Methods

### Ethical approval

The present study has been approved by the Graduate Studies Committee at the University of Baghdad, College of Veterinary Medicine, Baghdad, Iraq.

### Preparation of SAS

Sweet almond (*P. amygdalus*) seeds were purchased from a local market in Erbil, Iraq. They were mechanically ground using a grinder blender (Apex, Australia). Aqueous suspension of the ground almond was prepared at a concentration of 14.28%, as described previously [14].

### Animal housing

Albino Swiss origin mice (body weight 25-30 g) were used. They were housed with wood shavings as bedding at 21-25°C and had free access to water and pelleted rodent food. The light/dark cycle was 10/14 h.

### Induction of hyperlipidemia

Hyperlipidemia in mice was induced by adding 1% cholesterol to the diet along with 0.5% H_2_O_2_ to the drinking water, with *ad libitum* access to both food and water for 60 consecutive days [7,8,15].

### Animal grouping

Groups of mice (n=10/group) were treated as follows:

Group 1 (negative control) mice were provided a pelleted diet and drinking water *ad libitum* and treated with distilled water at 10 ml/kg/day orally using a gavage needle for 60 consecutive days.

Group 2 (positive control) mice were fed a hyperlipidemic diet with H_2_O_2_ in the drinking water for 60 consecutive days and then treated orally with distilled water at 10 ml/kg/day for 60 consecutive days.

Group 3 mice were provided the hyperlipidemic diet along with H_2_O_2_ in the drinking water for 60 consecutive days and then orally administered with SAS at a dose of 285 mg/kg/day for 60 consecutive days.

Group 4 mice were fed the hyperlipidemic diet and H_2_O_2_ in the drinking water for 60 consecutive days and then orally administered with SAS at a dose of 571 mg/kg/day for 60 consecutive days.

Group 5 mice were provided the hyperlipidemic diet and H_2_O_2_ in the drinking water for 60 consecutive days and then orally administered with SAS at a dose of 857 mg/kg/day for 60 consecutive days.

Group 6 mice were provided the hyperlipidemic diet and H_2_O_2_ in the drinking water for 60 consecutive days and then orally administered with SAS at a dose of 1128 mg/kg/day for 60 consecutive days.

Group 7 mice were fed the hyperlipidemic diet and H_2_O_2_ in the drinking water for 60 consecutive days and then orally administered with SAS at a dose of 1428 mg/kg/day for 60 consecutive days.

The doses of SAS were chosen based on our preliminary experiments and previous reports on mice [12,13,15].

### Blood sampling and biochemical measurements

At the end of the 60-day treatment regimen, blood samples were collected through cardiac puncture under anesthesia with ether [16]. Coagulation factors and lipid profile of the mice were determined. These included the following: Prothrombin time [17], partial thromboplastin time [18], clotting time [19], and platelet count [20]. Lipid profile was determined using an enzymatic method for the detection of total cholesterol (TC) and triglycerides (TG) using kits which were supplied by Linear Chemicals Company, Barcelona, Spain. High-density lipoprotein cholesterol (HDL-C) was determined as described previously [21], whereas low-density lipoprotein cholesterol (LDL-C) and very LDL-C (VLDL-C) were calculated using the Friedewald formula [22]. The absorbance wavelengths of the spectrophotometer (Apel Co. Ltd., Saitama, Japan) were set at 500 nm for TC and TG and 550 nm for HDL-C.

### Statistical analysis

Data were subjected to one-way analysis of variance followed by the least significant difference test [23]. p<0.05 was considered statistically significant.

## Results

Dietary cholesterol and H_2_O_2_ in the drinking water administered for 60 consecutive days induced hyperlipidemia in mice with concomitant changes in the blood coagulation factors (Table-1) and lipid profile (Table-2).

**Table-1:** The anticoagulant parameters of different groups of mice (controls and hyperlipidemic) treated with sweet almond suspension (SAS) in different doses daily for 60 consecutive days.

Platelet CountPlatelets ×10³/mm³	Clotting Time (min)	Partial thromboplastin time/sec	Prothrombin time/sec	Group
209.66±1.66^E^	2.88±0.12^C^	24.43±0.40^C^	9.14±0.04^C^	TA (negative control)-distilled water
572.83±7.86^A^	2.10±0.15^E^	19.56±0.26^E^	7.46±0.17^D^	TB (positive control)-distilled water
553.66±14.27^A^	2.00±0.14^E^	20.63±0.44^E^	7.52±0.17^D^	T1 (SAS 285 mg/kg, orally)
482.66±19.44^B^	2.51±0.10^D^	22.01±0.64^D^	7.52±0.15^D^	T2 (SAS 571 mg/kg, orally)
413.00±9.07^C^	2.85±0.13^C^	25.63±1.31^C^	9.88±0.49^C^	T3 (SAS 857 mg/kg, orally)
285.16±9.79^D^	3.19±0.08^B^	29.40±0.39^B^	13.05±0.39^B^	T4 (SAS 1128 mg/kg, orally)
255.16±7.95^D^	3.53±0.09^A^	31.86±0.48^A^	15.23±0.23^A^	T5 (SAS 1428 mg/kg, orally)

n=10 mice/group. Different letters in a column refer to significant differences among the groups (p<0.05). TB and T1-T5-hyperlipidemic mice. Values represent mean±SE

**Table-2:** The lipid profile of different groups of mice (controls and hyperlipidemic) treated with sweet almond suspension (SAS) in different doses daily for 60 consecutive days.

VLDL-C mg/dl	LDL-C mg/d	HDL-C mg/dl	TAG mg/dl	TC mg/dl	Group
15.90±0.34^E^	28.31±1.04^F^	56.30±1.70^B^	78.22±2.00^D^	100.77±1.02^F^	TA (negative control)-distilled water
37.27±0.55^A^	233.49±4.34^A^	41.85±0.79^D^	185.99±4.22^A^	312.38±2.91^A^	TB (positive control)-distilled water
36.30±0.38^A^	225.92±3.85^AB^	44.19±0.90^D^	179.18±3.98^A^	305.88±2.29^B^	T1 (SAS 285 mg/kg, orally)
29.25±0.59^B^	223.16±3.90^B^	48.63±0.71^C^	138.98±2.00^B^	299.50±2.25^B^	T2 (SAS 571 mg/kg, orally)
22.99±0.34^C^	166.95±3.34^C^	51.17±0.78^C^	115.00±1.75^C^	241.11± 3.00^C^	T3 (SAS 857 mg/kg, orally)
18.76±0.24^D^	89.52±2.34^D^	57.19±0.60^B^	93.81±2.25^D^	164.73±2.77^D^	T4 (SAS 1128 mg/kg, orally)
16.73±0.32^E^	48.30±1.98^E^	61.20±0.47^A^	84.62±1.76^D^	124.74±2.07^E^	T5 (SAS 1428 mg/kg, orally)

n=10 mice/group. Different letters in a column refer to significant differences among the groups (p<0.05). TB and T1-T5-hyperlipidemic mice. Values represent mean±SE

The results showed a significant increase in prothrombin time, partial thromboplastin time, and clotting time and a significant decrease in the platelet count after increasing doses of SAS (857, 1128, and 1428 mg/kg), especially at the dose rate of 1428 mg/kg, in comparison with the positive control group (Table-1). There were significant decreases in TC, triacylglycerol (TAG), LDL-C, and VLDL-C and significant increases in HDL-C after increasing the doses of SAS (857, 1128, and 1428 mg/kg) in comparison with the positive control group (Table-2).

## Discussion

In accordance with the previous studies [7,8,14], we supplied a dietary cholesterol supplement and H_2_O_2_ in the drinking water of mice for 60 consecutive days to induce hyperlipidemia. The changes in the coagulation parameters of the hyperlipidemic mice treated with SAS could be attributed to several properties of almonds. Arginine is one of the most abundant amino acids found in nut proteins and it is the precursor of nitric oxide, which plays an essential role in the inhibition of platelet aggregation [24]. Vitamin E is naturally found in almonds and it is thought to prevent platelet aggregation [25]. In addition, the folic acid in almonds helps to lower levels of homocysteine, the amino acid that is thought to contribute to the build-up of fatty plaques in arteries [26]. Linolenic acid may reduce the risk of coronary heart disease, possibly due to its anticlotting effects [27]. Our results are in agreement with another study, in which it was found that SAS has an anticoagulant activity due to its effect on coagulation factors [15].

Several factors may have contributed to the effects of SAS on the lipid profile in mice in the present study. Almond seeds are rich in fiber, which is recognized as a useful ingredient for controlling the oxidative process in food products; additionally, lipid-altering phytochemicals such as the plant sterols and saponins that are found in almond seeds may alter the lipid profile [28]. The major phytosterol component is β-sitosterol, which is one of several plant sterols that have been implicated in cholesterol-lowering effects [29]. Notably, Vitamin E is transported in the LDL particle and has been shown to reduce the risk of coronary heart disease [30]. This cardioprotective effect of Vitamin E appears to be due to its inhibition of LDL oxidation [30]. High-almond diets decrease the concentration of apolipoprotein B, a component of VLDL and LDL in the serum [31]. It has been observed that decreasing apolipoprotein B concentration while increasing the amount of almond in the diet decreases both the LDL-C concentration and the number of LDL particles [31]. The results of the present study on the lipid profile are consistent with those of a previous one, in which it was found that SAS has antihyperlipidemic activity through its effect on lipid profile by decreasing the level of TC, TAG, LDL-C, and VLDL-C and increasing that of HDL-C in rats [6]. Our study also supports a previous finding in diabetic rats, in which the alcoholic extract of sweet almond improved the metabolic derangement in the lipid profile and reduced lipid peroxidation in the serum [32].

## Conclusion

Sweet almond was found to exert anticoagulant and antihyperlipidemic effects in mice, with the best dose of SAS being 1428 mg/kg.

## Authors’ Contributions

LAK conceived and designed the experiment. AAT performed the experiment. LAK analyzed the data statistically. LAK and AAT contributed reagents/materials/animals/analysis tools. LAK wrote the paper. All authors read and approved the final manuscript.
